# Can advanced new radiation therapy technologies improve outcome of high grade glioma (HGG) patients? analysis of 3D-conformal radiotherapy (3DCRT) versus volumetric-modulated arc therapy (VMAT) in patients treated with surgery, concomitant and adjuvant chemo-radiotherapy

**DOI:** 10.1186/s12885-016-2399-6

**Published:** 2016-06-10

**Authors:** Pierina Navarria, Federico Pessina, Luca Cozzi, Anna Maria Ascolese, Francesca Lobefalo, Antonella Stravato, Giuseppe D’Agostino, Ciro Franzese, Manuela Caroli, Lorenzo Bello, Marta Scorsetti

**Affiliations:** Radiotherapy and Radiosurgery Department, Humanitas Cancer Center and Research Hospital, Milan, Italy; Department of Neurooncological surgery, Department of Medical Biotechnology and Translational Medicine, Università degli Studi di Milano and Humanitas Cancer Center and Research Hospital, Milan, Italy; Department of Neurosurgery, Fondazione IRCCS Ca’ Granda Ospedale Maggiore Policlinico, Milan, Italy; Istituto Clinico Humanitas Cancer Center, Via Manzoni 56 20089 Rozzano, Milano, Italy

**Keywords:** Glioblastoma, Volumetric modulated arc therapy, Conformal therapy

## Abstract

**Background:**

To assess the impact of volumetric-modulated arc therapy (VMAT) compared with 3D-conformal radiotherapy (3DCRT) in patients with newly diagnosed high grade glioma in terms of toxicity, progression free survival (PFS) and overall survival (OS).

**Methods:**

From March 2004 to October 2014, 341 patients underwent surgery followed by concomitant and adjuvant chemo-radiotherapy. From 2003 to 2010, 167 patients were treated using 3DCRT; starting from 2011, 174 patients underwent VMAT. The quantitative evaluation of the treatment plans was performed by means of standard dose volume histogram analysis. Response was recorded using the Response Assessment in Neuro-Oncology (RANO) criteria and toxicities graded according to Common Terminology Criteria for Adverse Event version 4.0.

**Results:**

Both techniques achieved an adequate dose conformity to the target. The median follow up time was 1.3 years; at the last observation 76 patients (23.4 %) were alive and 249 (76.6 %) dead (16 patients were lot to follow-up). For patients who underwent 3DCRT, the median PFS was 0.99 ± 0.07 years (CI95: 0.9–1.1 years); the 1 and 3 years PFS were, 49.6 ± 4 and 19.1 ± 3.1 %. This shall be compared, respectively, to 1.29 ± 0.13 years (CI95: 1.01–1.5 years), 60.8 ± 3.8, and 29.7 ± 4.6 % for patients who underwent VMAT (*p* = 0.02). The median OS for 3DCRT patients was 1.21 ± 0.09 years (CI95:1.03–1.3 years); 1 and 5 year OS was, 63.3 ± 3.8 and 21.5 ± 3.3 %. The corresponding results for 3DRCT patients were 1.56 ± 0.09 years (CI95:1.37–1.74 years), 73.4 ± 3.5, 30 ± 4.6 % respectively (*p* < 0.01). In both groups, prognostic factors conditioning PFS and OS were age, gender, KPS, histology and extent of resection (EOR).

**Conclusions:**

VMAT resulted superior to 3DCRT in terms of dosimetric findings and clinical results.

## Background

Surgery, followed by concomitant and adjuvant chemo-radiotherapy with temozolomide (TMZ), represents the standard of care for newly diagnosed glioblastoma (GB). However, survival remains poor with a median survival time and 3-year survival rate of 10–12 months and 6–8 % respectively [1]. In WHO grade III glioma the outcome in terms of progression free survival (PFS) and overall survival (OS) is better compared to GB patients but the incidence of local recurrence is still considerable, above all in the case of astrocytic tumors subtypes [2–5]. The pattern of recurrence is almost local [6] and often the inability to deliver a sufficient radiation dose to an adequate target volume has been considered as one of the leading cause of local failure [7, 8]. In order to improve local control and survival, a clearer identification of the volume at the greatest risk of recurrence is probably required, while the radiation exposure of healthy brain tissue should be reduced [9]. The close proximity of important normal brain structure, such as chiasma, optical nerve or brain stem, represents a limit to deliver the right dose to the right tumor volume. The recent availability of modern high-precision radiotherapy techniques [10–13] allows a more adequate delivery of high doses with maximum sparing of normal tissue. Intensity Modulated Radiation Therapy (IMRT) has being increasingly evaluated and exploited for the treatment of GB [14, 15]. Compared to 3D-conformal radiation therapy (3DCRT), IMRT provides similar results in terms of target coverage, but it results in a better dose conformity to the target with a better sparing of the organs at risk (OARs). RapidArc technology (Varian Medical Systems, Palo Alto, USA) is a currently available form of volumetric-modulated arc therapy (VMAT) that, with the variation of gantry rotation speed, beam shaping aperture and delivery dose rate, achieves an intensity-modulated dose distribution. The variation of the three dynamic parameters is used to cover the planning target volume (PTV) with clinically acceptable dose and to spare organs at risk and normal tissues, reducing the treatment time. However it is not yet clear if this new RT technology, compared with traditional 3DCRT, might reduce the acute and long-term neurotoxicity or could improve PFS and OS.

Based on the lack of evidence regarding the optimal radiation therapy technique for patients with newly diagnosed high grade glioma (HGG), the aim of this study was to evaluate the outcome of patients treated with different techniques in our institution in the last 10 years. Dosimetric and clinical findings in terms of PFS and OS were analyzed and compared.

## Methods

### Patients and procedures

The present retrospective study includes patients with newly diagnosed glioblastoma and WHO Grade III gliomas (anaplastic astrocytoma AA, anaplastic oligo-astrocytoma AOA and anaplastic oligo-dendroglioma AO) treated at our institute. All patients were treated in agreement with the Helsinki declaration. This study is a summary of a retrospective analysis to the treatment charts. The Humanitas Institute’s ethical committee does not require a formal approval in case of retrospective study in which a formal consent for handling patient medical data was obtained at the time of admission according to the deliberation of the national agency for clinical studies of 2008. All patients underwent surgery plus concomitant radiotherapy and TMZ chemotherapy followed by adjuvant TMZ according to the Stupp’s scheme [1].

Surgical resection was performed with the aid of intraoperative neurophysiological monitoring and brain mapping techniques with the aim to maximize resection respecting eloquent areas. Patients underwent preoperative baseline magnetic resonance imaging (MRI) studies, functional MR imaging (fMRI), and diffusion tensor imaging (DTI) with fiber tractography (DTI-FT). Volumetric scan analysis was used for defining tumor location and volume. Tumor volume was computed on volumetric postcontrast T1-weighted MRI scans. All patients underwent intraoperative neurophysiological brain mapping, when needed, and monitoring. The craniotomy was guided by neuronavigation system (Curve, BrainLab, Heimstemen, Germany) and tailored to expose the cortex corresponding to the tumor area. The lesion limits were defined with the aid of intraoperative pre-calibrated multifrequency (3.75–10 MHz) convex transducer ultrasound machine (UST-9120, footprint 20 mm, ProSound Alpha 7, Hitachi Aloka Medical Ltd,Zurich, Switzerland). Cortical mapping was performed to define the cortical safe-entry zone. Subcortical brain mapping was then continued, along with tumor resection. The extent of resection (EOR) was assessed on volumetric MRI studies obtained within 48 h after surgery by segmentation analysis (BrainLab Heimstemen, Germany). Complete resection (CR) was defined as residual tumor volume lower than 1 cm^3^, subtotal resection (SR) as residual tumor volume between 1 cm^3^ and 10 cm^3^ and partial resection (PR) as residual tumor volume greater than 10 cm^3^ [16]. Within 4 weeks from surgery, all patients underwent concurrent chemo-radiotherapy, consisting in 60Gy/2 Gy fraction with concomitant administration of oral TMZ (75 mg/m^2^), followed by adjuvant TMZ (200–250 mg/m^2^ for 5/28 days) for about 6–8 cycles. For all patients computed tomography (CT) scan and MRI were acquired for radiation therapy planning. MR imaging was performed on a 1–3T whole body system (Magnetom Verio, Siemens Medical Solutions, Erlangen, Germany). The standard protocol included pre-contrast T1-weighted fluid-attenuated inversion recovery imaging (FLAIR) and T2-weighted 3D-FLAIR followed by T1-weighted magnetization-prepared rapid gradient echo (MP-RAGE) images acquired after intravenous administration of 0.6 cm^3^/kg (1.0 mmol/ cm^3^) Gadolinium-based contrast agent.

### Target volume definition and planning optimization

To precisely delineate the extension of tumor and eventual residual tumor after surgery, pre- and post-operative enhanced T1-MRI and FLAIR-MRI sequences were used and co-registered. The procedure for target definition included post-contrast CT scan too. Patients were placed in supine position with arms close to the body. A personalized thermoplastic mask was used for patient immobilization and repositioning. All CT scans, extending from the top of the skull to the third cervical vertebrae, were acquired with 1–3 mm slice thickness and imported in the iPlan stereotactic treatment planning system (Brainlab Ag, Feldkirchen, Germany). An automatic rigid co-registration was performed for all patients. Clinical target volume (CTV) was generated by adding an isotropic margin of 10 mm to the abnormality on preoperative enhanced contrast T1-MRI. Attention was paid to include in the CTV the entire surgical cavity, the residual tumor, if present, and abnormality FLAIR area detected on postoperative MRI. Organs at risk delineated were brainstem, optic nerves, chiasm, lenses and healthy brain. Planning target volume (PTV) was defined as an isotropic expansion from CTV of 3 mm. We choose to use 1 cm of margin to generate CTV and 3 mm for PTV with the aim to reduce the risk of damage on healthy brain tissue. All plans were optimized on PTV. From 2003 to 2010 plans were processed using 3DCRT; plans were designed for multiple coplanar fixed gantry beams shaped with the multi-leaf collimator and delivered with Clinac 2100 linear accelerator (Varian Medical System, Palo Alto, USA). Starting from 2011 to 2014, all patients were treated with VMAT in the RapidArc form (Varian Medical System, Palo Alto, USA) using single or multiple full or partial coplanar or non-coplanar arcs based on an individualized plan optimization. Plans were optimized aiming to achieve a PTV coverage of D_95 %_ > 95 %. All patients were treated on a TrueBeam linear accelerator (Varian Medical System, Palo Alto, USA).

### Evaluation of treatment plans

The quantitative evaluation of the treatment plans was performed by means of standard dose volume histogram (DVH) analysis. A number of relevant metrics were scored and assessed for all target volumes and OARs. These included V_95_, V_107_, D_99 %_, homogeneity index (HI) defined as HI = (D_5 %_–D_95 %_)/D_mean_ and conformity index (CI) defined as reported in the RTOG1993 for the target volumes, together with a number of appropriate D_x %_ or V_xGy_ related to the OARs. Average DVH were computed over the two cohorts of patients for all target and OAR structures with a dose binning of 0.02Gy. Mean values of the selected parameters for the two cohorts of patients were considered.

### Outcome evaluation

Clinical outcome was evaluated by neurological examination and brain MRI performed 1 month after radiation therapy and then every 3 months. Recurrence was defined as follows: “in-field recurrence” (IFR) if it was overlapped with isodose of 95 %, “marginal recurrence” (MR) if it was overlapped with isodose of 90 %, “outfield recurrence” (OR) if it was outside of treatment volume and “distant recurrence” (DR) if it occurred in other brain lobe [17]. MRI images acquired during follow up were co-registered with pre-radiation therapy MRI to precisely define the site of relapse. Response was recorded using the Response Assessment in Neuro-Oncology (RANO) criteria [18]. Hematologic and non-hematologic toxicities were graded according to Common Terminology Criteria for Adverse Events version 4.0.

### Statistical analysis

Standard descriptive statistics (mean standard deviation and cross tabulation analysis) was used to describe the data general behavior. Survival and recurrence time observations were plotted according to the method of Kaplan and Meier, starting from the date of surgery. The log-rank test was used to carry out the univariate analysis, in order to investigate the prognostic role of individual variables. Multivariate Cox model was used as a method to estimate the independent association of a variable set with overall and progression free survival. The SPSS statistics package (IBM Corp,Armonk, USA), version 22, was used for the analysis.

## Results

### Patients and treatments

From March 2004 to October 2014, 341 consecutive patients referred to our institution for HGG were included in this analysis; 124 (36 %) were female and 217 (64 %) male with a median age of 59 years (range 19–82 years); 251 (73.6 %) patients had a diagnosis of GB, and 90 (26.4 %) patients WHO grade III glioma. All patients underwent surgery followed by concomitant and adjuvant chemo-radiotherapy. Complete surgical resection (CR) was obtained in 115 patients (34 %), subtotal resection (SR) in 41 patients (12 %), partial resection (PR) in 116 patients (34 %) and biopsy only in 69 patients (20 %). Incomplete resection or biopsy was performed in the case of tumors involving eloquent areas. 3DCRT was performed from 2003 to 2010 in 167 patients (49 %) and VMAT from 2011 to 2014 in 174 patients (51 %). The two cohorts, treated with different radiation therapy modality, 3DCRT vs VMAT, were homogeneous in terms of patients, tumors characteristics and treatments performed as shown in Table 1.

**Table 1:** Patients and treatment characteristics for the two groups

	3DCRT		VMAT	
	*Number*	*Percent*	*Number*	*Percent*
*Total*	*167*	*49*	*174*	*51*
*Gender*				
Female	57	34	67	38.5
Male	110	66	107	61.5
*Median Age*	53 yrs (range 20–82 yrs)		54 yrs (range 19–82 yrs)	
*Karnosky Performance Status (KPS)*				
60	6	3	3	2
70	26	16	25	14
80	68	41	52	30
90	44	26	52	30
100	23	14	42	24
*Histology*				
Glioblastoma (GBM)	129	77	122	70
Anaplastic Astrocytoma (AA)	20	12	29	17
Anaplastic Oligoastrocytoma (AOA)	9	5.5	10	6
Anaplastic Oligodendroglioma (AO)	9	5.5	13	7
*Isocitrate dehydrogenase (IDH) mutation status*				
Wild type	–	–	126	72
Mutated	–	–	38	22
NA (not available)	167	100	10	6
*1p19q codelation*				
Codeleted	18	11	30	17
Non codeleted	20	12	22	13
NE (not evaluated for GBM pts)	129	77	122	70
*O-6-methylguanine-DNA-methyltransferase (MGMT) promoter methylation status*				
Methylated	94	54	107	61
Unmethylated	76	46	67	49
*Surgery*				
Biopsy (B)	47	28	22	13
Partial Resection (PR)	58	35	58	33
Subtotal Resection (SR)	15	9	26	15
Complete Resection (CR)	47	28	68	39
*Adjuvant treatment*				
RT + concomitant CHT+ adjuvant CHT (TMZ)	167	100	174	100

*Abbreviations*: *3DCRT* 3dimensional conformal radiotherapy, *VMAT* volumetric modulated arc therapy, *RT* radiotherapy, *CHT* chemotherapy, *TMZ* Temozolomide

### Dosimetric analysis

**Fig. 1:**
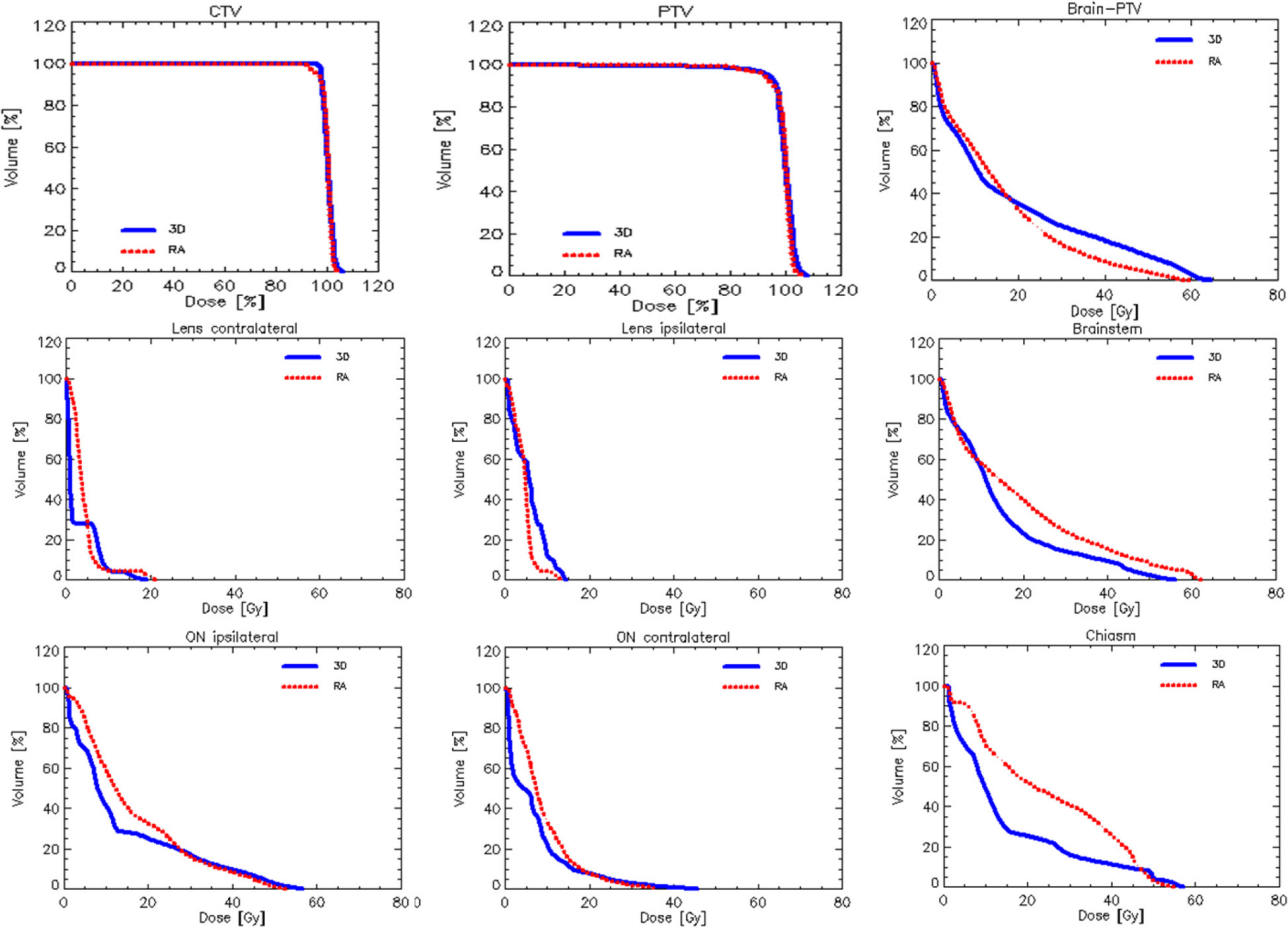
Mean DVH for CTV, PTV and OARs for 3DCRT and VMAT techniques

Results from the numerical analysis of the two cohorts of patients for the considered metrics relevant for CTV, PTV and the various OARs are reported in the Table 2. The target volumes were comparable in the two groups of patients: the mean PTV volume for the VMAT patients was 275 ± 112 cm^3^ and 203 ± 101 cm^3^ for the 3DCRT ones. The coverage objective was considered acceptable in all the cases. The percentage of VMAT plans fulfilling the V_95 %_ > 95 % objective shows an average V_95 %_ higher than the 3DCRT ones, where the under-dosage of the PTV mainly occurred in patients with PTV located near OARs. Table 2 shows that no significant over-dosage was observed with V_107 %_ = 0.47 and 0.09 cm^3^ for 3DCRT and VMAT respectively. VMAT technique allowed to achieve a better conformity index than 3DCRT simultaneously with an improvement of the homogeneity index. In both cases the difference was statistically significant. This result means that VMAT plans resulted in homogeneous irradiation of the target, at prescription dose while simultaneously improving the sparing of the surrounding healthy tissues compared to the older approach. Dose distribution can be visualized in the DVHs of Fig. 1, showing the PTV and CTV’s coverage and the OARs sparing. Concerning the OARs, it is interesting to focus on the healthy brain diagram. Medium-high doses (V_20Gy_ and V_35Gy_) are lower for the VMAT patients while in the low dose region (V_3Gy_ and V_5Gy_) 3DCRT plans give better results. Anyhow while the percentage deviations between VMAT and 3DCRT values were at maximum around 5 % for the V_3Gy_ in the low dose region, they increase up to around 11 % for the V_35Gy_ in the medium-high dose zone, showing a not negligible medium dose reduction for the VMAT plans. For what regards the other OARs (lenses, optic nerves, chiasm, eyes and brainstem), DVHs are obviously strongly affected by the relative localization of the lesions in the brain. VMAT technique allows to obtain in some cases, in particular for the ipsi-lateral OARs, a slightly reduction and control of the high doses. On the other side 3DCRT plans generally show a reduction of the mean doses as evidenced by the DVHs.

**Table 2:** Comparison of dosimetric results for 3DCRT and VMAT plans

	3DCRT	VMAT	*P*
PTV			
V_95 %_ (%)	93.6 ± 10.2 [52.5; 100.0]	97.2 ± 21.8 [32.4; 99.8]	ns
V_107 %_ (%)	0.5 ± 0.9 [0; 2.8]	0.1 ± 0.3 [0.0; 1.4]	ns
Vol (cm^3^)	203.1 ± 101.4 [91.2; 490.8]	274.8 ± 112.9 [99.6; 507.7]	ns
D_99 %_ (%)	85.4 ± 19.7 [4.37; 97.30]	82.8 ± 23.7 [16.8; 97.2]	ns
HI	0.13 ± 0.8 [0.04; 1.21]	0.09 ± 0.04 [0.03; 0.50]	0.04
CI	1.40 ± 0.30 [1.02; 2.09]	1.04 ± 0.02 [1.00; 1.09]	<0.01
Brain-PTV			
V_3Gy_(%)	75.0 ± 18.1 [42.0; 100.0]	79.9 ± 14.8 [51.9; 99.4]	ns
V_5Gy_ (%)	69.3 ± 18.1 [42.0; 100.0]	72.9 ± 16.3 [46.6; 98.0]	ns
V_20Gy_ (%)	35.4 ± 16.1 [14.2; 75.5]	32.6 ± 13.3 [13.9; 53.7]	ns
V_35Gy_ (%)	21.6 ± 12.6 [3.5; 48.9]	12.1 ± 5.6 [5.1; 24.1]	0.03
D_mean_ (Gy)	18.8 ± 7.0 [10.8; 66.4]	16.5 ± 4.8 [9.9; 24.9]	ns
D_1 %_ (Gy)	58.9 ± 5.1 [39.9; 65.3]	55.9 ± 1.8 [53.4; 59.3]	ns
Ipsilateral Lens			
D_1 %_ (Gy)	6.4 ± 4.5 [0.5; 14.9]	5.3 ± 2.8 [0.6; 13.9]	ns
Controlateral Lens			
D_1 %_ (Gy)	3.6 ± 4.9 [0.4; 19.8]	5.1 ± 4.2 [0.7; 21.1]	ns
Ipsilateral Optic Nerve			
D_1 %_ (Gy)	19.6 ± 19.2 [1.0; 57.2]	25.5 ± 18.2 [0.9; 53.7]	ns
Controlateral Optic Nerve			
D_1 %_ (Gy)	10.1 ± 11.7 [0.7; 47.2]	12.9 ± 8.9 [1.3; 36.6]	ns
Chiasm			
D_1 %_ (Gy)	22.2 ± 18.1 [1.4; 56.7]	32.0 ± 18.6 [1.8; 55.9]	0.02
Brainstem			
D_1 %_ (Gy)	33.5 ± 18.4 [1.7; 57.3]	40.9 ± 18.0 [4.9; 62.5]	0.04

### Outcome of patients

Relatively to the entire cohort of patients, 202 (59 %) presented neurological symptoms before adjuvant chemo-radiotherapy and 139 (41 %) patients were asymptomatic. Clinical remission, complete or partial, was obtained in the vast majority of patients 198/202 (98 %) after treatment. Neurological status worsened in 4 (2 %) patients. Patients evaluable for the actuarial analysis were 325 because 16 were not available to follow up. Among evaluable patients, 215/325 (66 %) relapsed. Recurrence at the same site of treatment occurred in 187 (57.5 %) and in 18 of these, it was associated with a distant tumor progression. Eighteen (5.5 %) patients had only distant progression. In relation to the type of RT performed, 3DCRT or VMAT, recurrences occurred in 114/160 (71 %) and 101/165 (61 %) patients respectively. The recurrence was local in 94 (82.5 %), local and distant in 6 (5.2 %) and distant in 14 (12.3 %) for 3DCRT patients, while for VMAT patients, it was local for 74 (73.2 %), local and distant for 12 (11.8 %) and distant for 15 (15 %). Details about local site of relapse, “in-field” (IFR), “marginal” (MR) or “out-field” (OFR) are shown in Table 3. No out-field recurrence was recorded in our series.

**Table 3:** Site of recurrence characteristics in relation to different type of treatments performed 3DCRT or VMAT

	3DCRT 160		VMAT 165	
	*Number*	*Percent*	*Number*	*Percent*
Recurrence	114	71	101	61
Local (L)	94	82.5	74	73.2
Local + Distant(L + D)	6	5.2	12	11.8
Distant(D)	14	12.3	15	15
In field (IF)	97	97	82	95.4
Marginal(M)	3	3	4	4.6
Outfield (OF)	0	0	0	0

*Abbreviations*: *3DCRT* 3dimensional conformal radiotherapy, *VMAT* volumetric modulated arc therapy

The median follow up time was 1.3 years (range 0–11.5 years) for the entire cohort, 1.2 years (range 0–11.5) for the 3DCRT group and 1.4 years (range 0–5.1) for the VMAT one respectively. At the last observation time 76 patients (23.4 %) were alive and 249 (76.6 %) died.

**Fig. 2:**
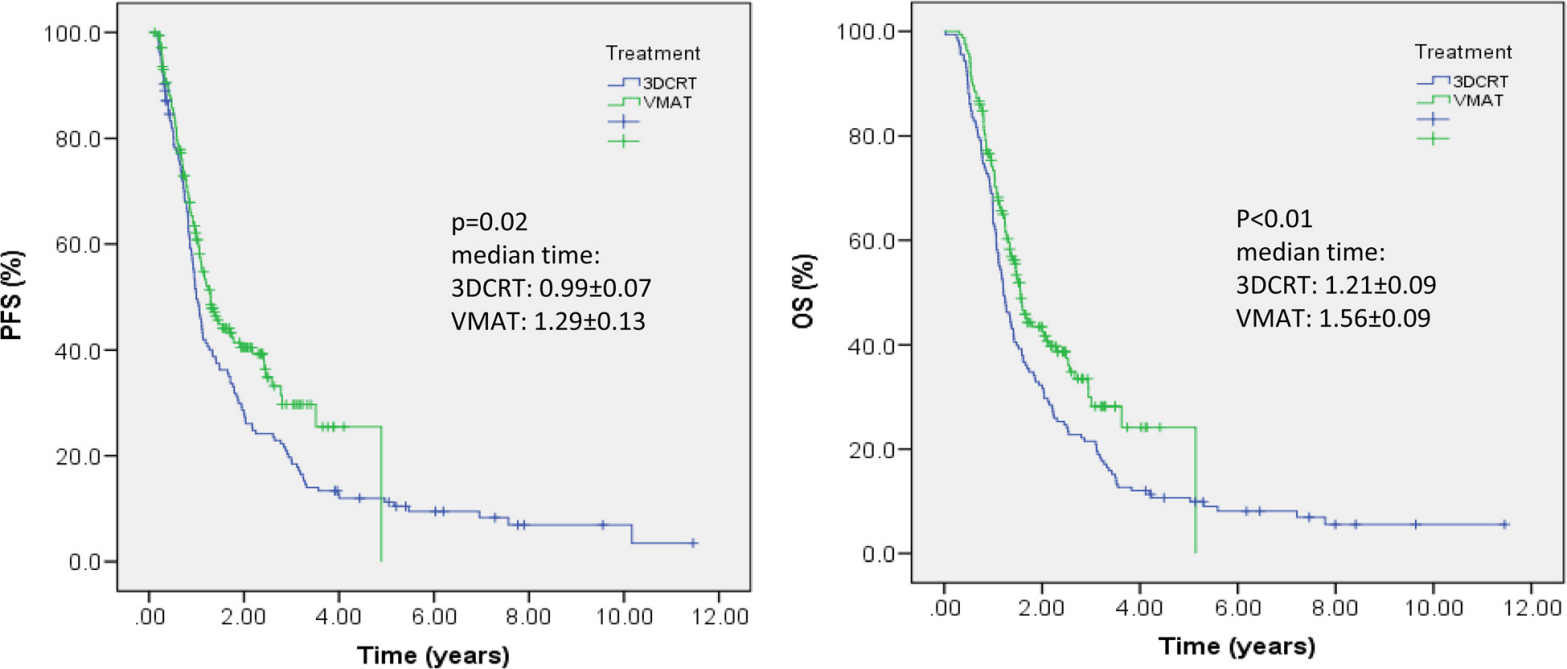
Progression Free Survival (PFS) and Overall Survival (OS) in patients with diagnosed High grade Gliomas (HGG) treated with two different radiation therapy techniques: 3Dimensional conformal radiation therapy (3DCRT) or Volumetric Modulated Arc Therapy (VMAT)

Table 4 summarizes the actuarial data for the 3DCRT and VMAT cohorts relatively to overall survival and progression free survival. For both observables, the two groups resulted significantly different (*p* = 0.02 for PFS and *p* < 0.01 for OS) as it results also from Fig. 2.

**Table 4:** Summary of median and 1–3–5 years actuarial data for overall survival and progression free survival for the 3DCRT and VMAT cohorts

	Median time [years]	*p*	1-year	3-years	5-years
Progression free survival					
3DCRT	0.99 ± 0.07 (CI95:range 0.9–1.1)	0.02	49.6 ± 4 %	19.1 ± 3.1 %	11.2 ± 2.5 %
VMAT	1.29 ± 0.13 (CI95:range 1.01–1.5)		60.8 ± 3.8 %	29.7 ± 4.6 %	29.7 ± 4.6 %
Overall survival					
3DCRT	1.21 ± 0.09 (CI95:1.03–1.3)	<0.01	63.3 ± 3.8 %	21.5 ± 3.3 %	10.6 ± 2.5 %
VMAT	1.56 ± 0.09 (CI95:1.37–1.74)		73.4 ± 3.5 %	30 ± 4.6 %	24.2 ± 5.4 %

On univariate analysis, age, gender, Karnofsky performance scale (KPS), histology and EOR were clinical variables which influence PFS and OS in both groups, 3DCRT and VMAT. Particularly patients with age <70 years, KPS >80, WHO grade III glioma versus GB, and undergoing CR/SR, had a better outcome. About biological markers, isocitrate dehydrogenase 1 (IDH1) status was available only for patients treated in the last years and co-deletion were performed only for non GB patients, the minority of our patients and therefore no correlation were made between the 2 groups. In relation to *O-6-methylguanine-DNA-methyltransferase (MGMT) promoter* status, it resulted statistically significant for both groups; patients with methylated MGMT had better outcome compared to un-methylated ones. Details are shown in Table 5. In multivariate analysis only age did resulted significantly associated to differences in OS or PFS. All other prognostic factors, significant in univariate, resulted significant also in the multivariate regression (with *p* < 0.01) for both 3DCRT and VMAT cohorts.

**Table 5:** Prognostic factors in univariate analysis for progression free survival (PFS) and Overall Survival (OS). Data reported are the median survival time for the various groups and the statistical significance

	3DCRT	*p*	VMAT	*p*
Overall survival				
Age^a^	a: 1.33 ± 0.09 b: 1.06 ± 0.19	0.04	a: 1.59 ± 0.23 b: 1.25 ± 0.21	0.12
KPS^b^	a: 1.06 ± 0.50 b: 2.03 ± 0.53	<0.001	a: 1.03 ± 0.09 b: 2.95 ± 0.51	<0.001
Histology^c^	a: 3.11 ± 0.60 b: 1.11 ± 0.06	<0.001	a: 3.62 ± 0.39 b: 1.25 ± 0.09	<0.001
EOR^d^	a: 1.82 ± 0.31 b: 1.02 ± 0.04	<0.001	a: 2.68 ± 0.44 b: 1.19 ± 0.12	<0.001
MGMT^e^	a: 1.19 ± 0.06 b: 1.48 ± 0.45	0.002	a: 1.23 ± 0.13 b: 1.99 ± 0.27	0.009
Progression free survival				
Age^a^	a: 1.10 ± 0.11 b: 0.82 ± 0.25	0.02	a: 1.37 ± 0.24 b: 0.97 ± 0.08	0.06
KPS^b^	a: 0.86 ± 0.04 b: 2.01 ± 0.44	<0.001	a: 0.83 ± 0.09 b: 2.78 ± 0.59	<0.001
Histology^c^	a: 2.77 ± 0.57 b: 0.93 ± 0.05	<0.001	a: 3.51 ± 0.42 b: 0.99 ± 0.09	<0.001
EOR^d^	a: 1.68 ± 0.34 b: 0.83 ± 0.06	<0.001	a: 2.58 ± 0.5 b: 0.92 ± 0.16	<0.001
MGMT^e^	a: 0.95 ± 0.04 b: 1.40 ± 0.36	0.02	a: 0.98 ± 0.12 b: 1.70 ± 0.44	0.02

*Abbreviations*: *3DCRT* 3 dimensional conformal radiotherapy, *VMAT* volumetric modulated arc therapy

^a^a:<70 or b:>70 years

^b^a:<80 vs b:>80

^c^a: Grade III glioma(anaplastic astrocytoma, Anaplastic oligodendroglioma, anaplastic oligoastrocytoma) vs b: Glioblastoma

^d^a: Gross total resection vs b: Partial resection or biopsy

^e^a: MGMT negative or b: positive

### Toxicity

Perioperative complications occurred in 30 (9.6 %) patients. Immediate neurological deficits were found in 22 (6.4 %) patients, in 11 cases recovered within 1 months. No perioperative mortality occurred. All patients were evaluated for toxicity during RT with concomitant TMZ and during the adjuvant chemotherapy treatment time. All patients completed the scheduled RT planning. No severe hematologic toxicity was recorded during treatment. During adjuvant chemotherapy 9 patients (2.7 %) had grade III thrombocytopenia, 21 (6.4 %) patients grade III neutropenia, 6 (1.8 %) patients grade III anemia and 9 (2.7 %) patients grade IV hematologic toxicity. Chemotherapy was interrupted in 6 (1.8 %) patients, and delayed or reduced in 14 (4.3 %) patients. A moderate to severe fatigue occurred in 48 (15 %) patients. Six patients had a deep venous thrombosis and 9 a severe lung infection (pneumonia) resolved with medical therapy. No symptomatic radio-necrosis has been reported. No differences were recorded in patients treated with 3DCRT vs those treated with VMAT.

### Treatment at progression

Salvage treatment was performed in 122/215 (56.7 %) patients and consisted in surgery alone in 16, radiation therapy alone in 6, and second line chemotherapy alone in 80 patients. A combined treatment was performed in 20 patients; surgery plus chemotherapy in 8, RT plus chemotherapy (CHT) in 7, and surgery plus RT followed by CHT in 5 patients. At the last observation time, 30/57 (52.7 %) patients are died and 27/57 (47.3 %) are alive. Concerning the two groups of patients, salvage treatment was performed in 56/112 3DCRT patients and in 66/103 VMAT patients. It consisted in surgery alone in 8, RT alone in 3, CHT alone in 38, and combined treatment in 7 patients for the 3DCRT group; for the VMAT group, surgery alone was performed in 6 patients, RT alone in 3, CHT alone in 42, and combined treatment in 15. In both groups the chemotherapeutic agents more frequently used have been fotemustine and temozolomide, (93 and 87 % of cases respectively).

## Discussion

Radiotherapy is usually delivered with 3D-conformal techniques but several improvements in the technologies led to more complex delivery technique (IMRT and VMAT) used to treat different types of solid tumors with a considerable outcome improvement, allowing to deliver dose with excellent conformation around the tumor and a better sparing of normal structures [14, 15]. Even though IMRT is now considered a feasible and practical delivery technique, to date it is not the standard radiotherapy approach in the treatment of HGG considering that the most of RT department treat patients using 3DCRT, and many issues are still to be addressed.

Several studies, comparing 3DCRT and IMRT, were published, with evidence of similar results in terms of target coverage, dose homogeneity [19–27] and dose conformity. In the majority of the published papers, an overall benefit using IMRT was recorded but mainly, differences are minimal, without statistically relevance. Conversely, a greater sparing of OARs using IMRT has been highlighted, although to an extent that varies considerably from study to study.

The main issue is represented by the integral dose to healthy brain tissue, which it is still an open question in relation to different methods used to define the region of interest (i.e. whole brain-PTV *vs* whole brain-GTV or other), the analyzed dosimetric values and the kind of utilized technique (fixed-field IMRT, helical tomotherapy or VMAT).

While several experiences comparing fixed-field IMRT and 3DCRT are available, to our knowledge, only 3 studies comparing VMAT and 3DCRT were published so far. Wagner [19] on 14 consecutive glioma patients, showed a better target coverage in case of PTV close to OARs using IMRT compared to both VMAT and 3DCRT; the only convenience of using VMAT was a shorter treatment time, less monitor units and a small V107%. Zach [24] and similarly Shaffer [20] found that VMAT provided the best homogeneity coefficient with respect to 3D-CRT and IMRT (*p* < 0.0003 for all comparison), a better reduction of mean and maximum dose to OARs and healthy brain.

Literature data report mainly an equivalence from the dosimetric point of view between VMAT and 3DCRT technique for the GB treatments. From our data, VMAT appears adequate for the treatment of GB. Nevertheless this study is not a direct comparison between two techniques, since all the patients were not re-planned and compared with 3DCRT and VMAT, it clearly emerges that VMAT allows a higher level of control on the high doses, as expected from this technique. It allows to reduce the high doses to the OARs, while the 3DCRT plans decrease the contribution of the low doses, as it is clearly evidenced on the healthy brain.

Obviously, dosimetric data are strictly connected to the localization of the lesions, the proximity to the OARs and the adopted technique. A further interesting step might be a study on this group of patients analyzing the results in relation to the anatomical localization of the lesion (frontal, temporal and so on). To our knowledge no clinical studies comparing 3DCRT and VMAT are available.

The main issue is, in our opinion, if the technological advances could lead a real benefit in the local control and patients overall survival. For this reason we wanted evaluate the impact on PFS and OS in patients with diagnosed HGG treated in our institution in the last 10 years with *2* different RT modalities. We know that ours is a retrospective study comparing different radiotherapy techniques used to treat HGG patients over a fairly long period of time with the well know limits of this one. Besides this is one of the larger cohort concerning this issue and some findings recorded should be promising. The 2 groups evaluated were homogeneous for patients, tumor characteristics, therapeutic strategies used at diagnosis and recurrence. In fact, all patients underwent combined treatment and were treated with the same salvage treatment at recurrence. The only difference was the different RT technique used in the 2 group of consecutive patients. The results were highly satisfactory; in fact the 5 years OS observed was about 10 % in 3DCRT vs 24 % in VMAT group (*p* < 0.01).

In addition, the same procedure to identify the treatment tumor volume was used and we choose to utilize a limited margin to generate CTV and PTV compared to other experience with the aim to spare normal brain tissue as much as possible. Our strategies had proved to be safe and effective for the low rate of marginal recurrences (7/215 recurrences) and for the absence of outfield relapses.

In relation to the different RT techniques used, the following issues require comment. i) the reduction of medium-high doses to healthy brain tissue and OARs might be particularly relevant for the possibility to decrease acute and late neurotoxicity influencing quality of life, above all in long surviving patients. In addition, the reduction of neurotoxicity could allow a dose escalation, an employment of hypo-fractionated regimens or in case of disease recurrence, a second course of RT with lower risk of side effects; ii) the use of VMAT employed a significantly reduction of treatment time in relation to the high dose rate used with better patients compliance; iii) a comparable toxicity was recorded using VMAT, notwithstanding a greater volume received low doses compared to 3DCRT; particularly no interruption of treatment was needed and no severe subacute toxicity was observed; iv) however, these clinical findings have to be considered with caution for the lack of experience published so far, and for the retrospective nature of our studies; moreover there seems to be no reasons against the use of VMAT in the treatments of HGG.

## Conclusion

VMAT is superior in terms of dose conformity and sparing of the healthy brain at medium to high doses compared to 3DCRT. In this issue the availability of modern RT technique that permit a better conformity on the tumor with maximum sparing of normal organ and tissue could be change the outcome of these patients. There is no reason against the use of this technique in GB patients.

## Abbreviations

3DCRT, three dimensional conformal radiotherapy; AA, anaplastic astrocytoma; AO, anaplastic oligo-dendroglioma; AOA, anaplastic oligo-astrocytoma; CHT, chemotherapy; CI, conformity index; CR, complete resection; CT, computed tomography; CTV, clinical target volume; DR, distant recurrence; DVH, dose volume histogram; EOR, extent of resection; FLAIR, fluid-attenuated inversion recovery imaging; fMRI, functional magnetic resonance imaging; GB, glioblastoma; GTV, gross tumor volume; HGG, high grade glioma; HI, homogeneity index; IFR, in field recurrence; IMRT, intensity modulated radiation therapy; IODG1, isocitrate dehydrogenase 1; KPS, Karnofsky performance scale; MGMT, O-6-methylguanine-DNA-methyltransferase promoter; MP-RAGE, magnetization-prepared rapid gradient echo; MR, marginal recurrence; MRI, magnetic resonance imaging; OAR, organs at risk; OR, outfield recurrence; OS, overall survival; PFS, progression free survival; PR, partial resection; PTV, planning target volume; RANO, response assessment in neuro-oncology; RT, radiotherapy; TMZ, temozolomide; VMAT, volumetric modulated arc therapy; WHO, World health organization
